# Centrin-2 (Cetn2) mediated regulation of FGF/FGFR gene expression in *Xenopus*

**DOI:** 10.1038/srep10283

**Published:** 2015-05-27

**Authors:** Jianli Shi, Ying Zhao, Tyson Vonderfecht, Mark Winey, Michael W. Klymkowsky

**Affiliations:** 1 Molecular, Cellular & Developmental Biology University of Colorado Boulder, Boulder, Colorado 80309

## Abstract

Centrins (Cetns) are highly conserved, widely expressed, and multifunctional Ca^2+^-binding eukaryotic signature proteins best known for their roles in ciliogenesis and as critical components of the global genome nucleotide excision repair system. Two distinct Cetn subtypes, Cetn2-like and Cetn3-like, have been recognized and implicated in a range of cellular processes. In the course of morpholino-based loss of function studies in Xenopus laevis, we have identified a previously unreported Cetn2-specific function, namely in fibroblast growth factor (FGF) mediated signaling, specifically through the regulation of FGF and FGF receptor RNA levels. Cetn2 was found associated with the RNA polymerase II binding sites of the Cetn2-regulated FGF8 and FGFR1a genes, but not at the promoter of a gene (BMP4) whose expression was altered indirectly in Cent2 morphant embryos. These observations point to a previously unexpected role of Cetn2 in the regulation of gene expression and embryonic development.

Centrins (Cetn) are calmodulin-like eukaryotic signature proteins^1^. Cetn2-like and Cetn3-like subclasses of Cetns have been identified^2^,^3^. In the yeast *Saccharomyces cerevisiae* there is a single Cetn3-like *Cetn* gene, *CDC31;* its function is required for spindle pole body duplication^4^. The ciliated protozoa *Tetrahymena thermophila* contains (at least) four *Cetn* genes, three of which are expressed^5^.

Loss of either the Cetn2-like *Cen1* or the Cetn3-like *Cen2* genes produce non-redundant defects in basal body and cilia formation. While the Cetn2-like gene is essential for cell division, the Cetn3-like gene is not; cells null for the Cetn3-like *Cen2* gene appear to divide normally but have aberrant basal body organization^5^,^6^,^7^.

The roles of Cetns in vertebrate cells appear to be more subtle and diverse. Mice have four distinct *Cetn* genes; *Cetn2, Cetn3,* and *Cetn4* are typical intron containing genes, while *Cetn1* lacks introns and is thought to have been generated by a retrotransposition event from *Cetn2*^8^. Humans appear to lack a *Cetn4*-like gene^9^. Null mutations in mouse *Cetn1* lead to infertility apparently due to defects in sperm development^10^ Zebrafish *Cetn2* morphants^11^ and mice homozygous for a null mutation in *Cetn2*^12^ have no reported cell division phenotypes but display ciliopathy-related phenotypes. *Cetn3* and *Cetn4* null phenotypes in the mouse have not, to our knowledge, been reported.

The removal of all three *Cetn* genes has been achieved in the chick hyper-recombinogenic DT40 cell line. *Cetn2*, *Cetn3*, and *Cetn4* DT40 null cells display ***no*** apparent defects in centrosome formation or cell division but were hypersensitive to UV irradiation^13^. The radiation-sensitive phenotype observed in these cells was expected given the role of Cetn2 as an integral component of the nucleotide excision repair/xeroderma pigmentosum group C (XPC-RAD23-CETN2) complex^14^,^15^. Araki *et al*. reported that “almost 100% of CEN2 (sic) in the cell extract was co-precipitated with the anti-XPC antibody”^14^. While Cetns are certainly associated with centrosomes and basal bodies, the majority of cellular Cetn2 is apparently soluble and located throughout the cytoplasm and nucleus^16^. A number of other functions have been ascribed to Cetns, from the transport of heterotrimeric transducin G-proteins in retinal photoreceptor cells to interactions with heat shock proteins, the regulation of proteosome activity, and the nuclear transport of RNAs and proteins^3^. It is clear that Cetn2 and Cetn3 have distinct functional roles in a number of systems.

## Results and Discussion

The idiosyncratic aspects of *X. laevis* development can reveal gene functions hidden in other organisms^17^. We therefore set out to explore the roles of Cetns in early *X. laevis* development. Both *X. laevis and X. tropicalis* have multiple centrin genes, based on data accessed through Xenbase^18^. The gene/protein originally designated as Centrin (*X. laevis*: NCBI Reference Sequence: NP_001081398.1 and *X. tropicalis*: NP_001016387.1) or Centrin-1 (*X. laevis*: NCBI Reference Sequence: NP_001080127.1) display a Cetn2-like, rather than a Cetn1-like, genomic structure (see supplementary figure 1); we therefore refer to them as *Cetn2* (see below). No *Cetn1*-like gene appears to be present in either *X. laevis* or *X. tropicalis* genomes. The *Cetn3* and *Cetn4* genes identified in *X. laevis* are similar in genomic structure to those found in mouse and human.

**Figure 1:**
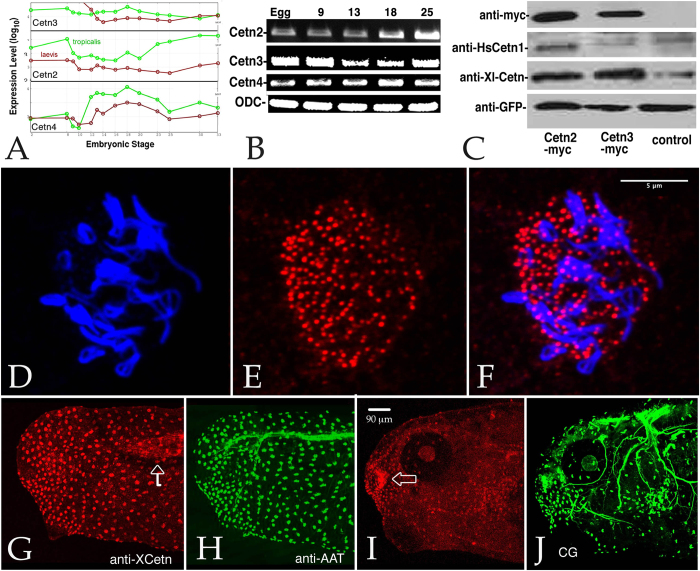
**A**: All three Cetn RNAs are present throughout the course of early development (data derived from Yanai *et al.* (2011)). **B**: This result was confirmed by RT-PCR analyses of Cetn2a, Cetn3l, and Cetn4 RNAs using ornithine decarboxylase (ODC) as a normalization control (embryonic stages are noted). **C**: Embryos injected with RNA (200 pg) encoding GFP alone or together with Cetn2a-myc or Cetn3l-myc were harvested at stage 11 and analyzed by SDS-PAGE-immunoblot. The anti-human Cetn1 antibody reacted preferentially with *X. laevis* Cetn2, while the anti-XlCetn antibody reacted with Cetn2 and Cetn3, as well as Cetn4 (data not shown). Ectodermal explants were fixed when sibling control embryos reached stage 18 and stained with anti-acetylated α-tubulin (AAT)(**D**) and anti-XlCetn antibodies (**E**; **F** displays the overlap of images in parts **D** and **E**); this revealed the localization of Cetns to the basal body region of cilia. A similar analysis was carried out on whole embryos **(G,H** - stage **25**, **I,J**-stage **35**) stained with anti-XlCetn (**G,I**) and anti-acetylated α-tubulin (**H,J**). Anti-Cetn staining of the myotome (arrow in part **G**) and Cetn’s localization to the olfactory region of the later stage embryo (arrow in part **I**) was obvious, as was its absence from the cement gland (“CG” in part **J**). Scale bar in part **F** marks 5 μm in parts **D-F**, scale bar in part **I** marks 90 μm in parts **G-J**.

The latest version of the *X. laevis* genome (7.1 as searched through the Xenbase Blast function in March 2015) reveals two distinct *Cetn2 (Cetn2a and Cetn2s)* and *Cetn3 (Cetn3l and Cetn3s)* genes, and apparently a single *Cetn4* gene. Our studies focus on the *Cetn2a*, *Cetn3l*, and *Cetn4* genes. Cetn2a corresponds to the 172 amino acid polypeptide labeled cetn1 or centrin (see above); Cetn3l corresponds to the 167 amino acid polypeptide labeled Cetn3 (GenBank: AAI29791.1). We isolated full length cDNAs that correspond to Cetn2a, Cetn3l, and Cetn4. An analysis of gene expression during early *Xenopus* embryogenesis by Yanai *et al.*^19^ (Fig. 1A) and our own RT-PCR data in *X. laevis* (Fig. 1B) indicate that *Cetn2a*, *Cetn3l*, and *Cetn4* RNAs are supplied maternally and are present at high levels throughout early development; we have not directly examined the expression levels of the *Cetn2s* or *Cetn3s* genes.

We used two different antibodies to localized Cetn proteins in *X. laevis*. The first is a rabbit antibody (anti-XlCetn)^20^ that recognizes Cetn2, Cetn3, and Cetn4 proteins based on its recognition of Cetn2a, Cetn3l, and Cetn4 polypeptides expressed from injected RNAs (Fig. 1C and data not shown). The second antibody is a commercially available rabbit antibody (anti-HsCetn1) that reacts preferentially with *X. laevis* Cetn2a compared to Cetn3l (Fig. 1C). Because the anti-HsCetn1 antibody produced higher overall background labelling, we used the anti-XlCetn antibody for most staining studies. Both anti-Cetn antibodies stain the basal body region of epidermal ciliated cells (Fig. 1D–F). There is also discernible staining of the myotome; neuronal microtubules are not stained (Fig. 1G–J - anti-XlCetn staining shown). Basal body localization of all three Cetns was confirmed using C-terminally GFP tagged forms of Cetn2a, Cent3l, and Cetn4 expressed from injected RNAs (see below).

**Figure 2:**
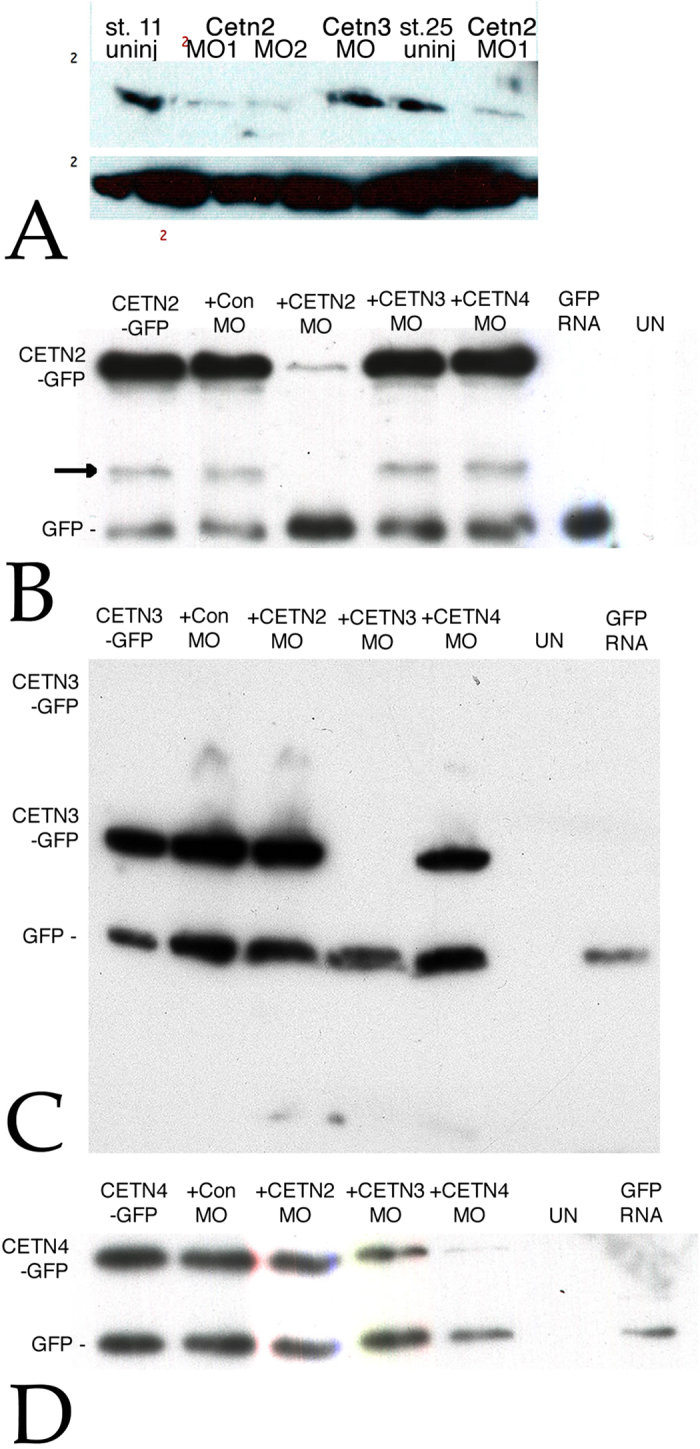
**A**: Embryos were injected into both cells at the two cell stage with RNAs encoding GFP (150 pgs per embryo) either alone or together with Cetn2MO1, Cetn2MO2, or Cetn3MO (10 ngs/side, 20 ngs total per embryo); at stage 11 or 25 the embryos were analyzed by SDS-PAGE and immunoblot using the anti-Human Cetn-1 antibody (which reacts preferentially with Cetn2 compared to Cetn3. There was a clear decrease in Cetn2 protein levels, persisting through stage 25. To confirm the specificities of the Cetn MOs both blastomeres of two cell embryos were injected with RNAs encoding GFP (200 pg/side) and RNAs encoding Cetn2a-GFP (**B**), Cetn3l-GFP (**C**), or Cetn4-GFP (**D**) RNAs with (“+”) or without Cetn MO (10 ng/side). These Cetn RNAs contain the target sequence of the corresponding morpholino. In addition, uninjected (“UN”) and embryos injected with GFP RNA alone were examined as controls for antibody specificity. Injected embryos were harvested at stage 11. Immunoblot analyses were carried out using an anti-rabbit GFP antibody. An apparent breakdown product of the Cetn2-GFP construct is indicated by the arrow in the Cetn2 MO panel.

To down-regulate the levels of specific Cetn proteins in embryos we commissioned Gene-Tools LLC to design anti-sense translation blocking modified DNA oligonucleotides (morpholinos or MOs) specific for *Cetn2a*, *Cetn3L*, and *Cetn4* RNAs (see supplemental figure. 2). The *Cetn2s* gene encodes a 201 amino acid long polypeptide that differs from Cetn2a primarily by the presence of a 29 amino acid insertion at its N-terminus. Similarly the *Cetn3S* gene encodes a 212 amino acid long polypeptide that differs from Cetn3l primarily by the presence of a 32 amino acid insertion at its N-terminus. Given the dramatic nature of the Cetn2 morphant phenotype (see below) we commissioned a second, completely non-overlapping anti-Cetn2 morpholino (Cetn2-MO2). The translation of the Cetn2s RNA is not expected to be altered by either of the Cetn2a morpholinos used in our studies; similarly expression of Cetn3s is not expected to be altered by the Cetn3l morpholino. Because of its dramatic nature, we concentrate the studies described here on the characterization of the Cetn2a morphant phenotype. Plasmids that encode versions of Cetn2a-GFP, Cetn3l-GFP, and Cetn4-GFP that contain sequences that match the Cetn2a-MO1, Cetn3l-MO, and Cetn4-MO morpholinos perfectly were created for specificity and rescue studies. The Cetn2-MO1 and Cetn2-MO2 morpholinos reduced the level of Cetn2 protein but not Cetn3 or Cetn4, while the Cetn3 and Cetn4 morpholinos specifically reduced the accumulation of their targets (Fig. 2).

In ectodermal explants, a single Cetn morpholino alone (10 ngs/embryo) did not dramatically or reproducibly disrupt the formation of cilia in multiciliated cells; cilia formation did appear to be disrupted when multiple Cetn morpholinos were used together (supplemental figure. 3 and data not shown). Cetn2 morphants exhibited embryonic phenotypes unlike those associated with a typical ciliopathy; they appeared similar to those associated with defects in FGF-mediated mesoderm formation^21^. This phenotype was distinct from that displayed by Cetn3 and Cetn4 morphants (supplemental figure. 3 and data not shown) as well as in Chibby (Cby) morphants (Cby is a basal body protein associated with the regulation of Wnt signaling)^22^.

**Figure 3:**
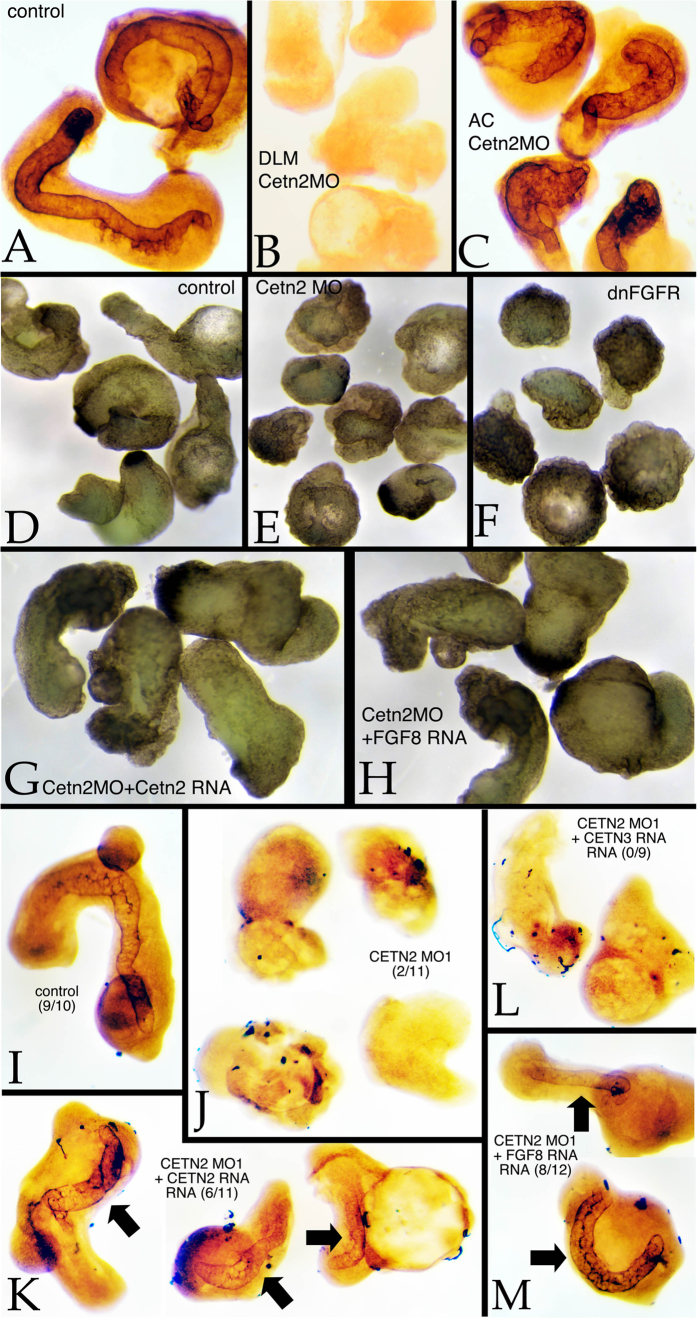
Animal cap/dorsal axial mesodermal zone (AC/DAMZ) explants were prepared from experimentally manipulated embryos when control (intact) embryos had reached stage 25. In wild type explants (A) staining with the anti-keratan sulfate antibody MZ15 revealed explant elongation and notochord formation. Both were absent in wild type AC/Cetn2 morphant DAMZ explants (B). Notochord formation occurred in Cetn2 morphant AC/wild type DAMZ explants (C). A comparison of dorsal axial mesoderm explant morphology (D-H) revealed the elongation of control explants (D), this elongation phenotype was absent in Cetn2 morphant explants (E) and dominant-negative FGFR RNA injected explants (F). In Cetn2 morphant explants, the elongation phenotype was rescued by either Cetn2-GFP (G) or FGF8 (H) RNA injection (200 pgs/embryo). Morpholinos were injected at 10 ng/embryo. Staining with MZ15 revealed the presence of notochordal tissues in control (I) explants, its absence in Cetn2 morphant explants (J), and its reappearance in Cetn2 RNA (K) and FGF8 (M), but not in Cetn3 RNA (L) injected Cetn2 morphant explants - number of explants with notochord staining per total number of explants is presented in brackets in panels I-M.

To test whether Cetn2 morpholinos disrupted FGF signaling, we used ectodermal and mesodermal explants, as described previously^23^. In culture, such explants normally elongate and form notochordal tissue within the mesodermal domain (Fig. 3A). When the mesodermal domain was taken from a Cetn2 morphant embryo, elongation was inhibited and notochordal tissue failed to form (Fig. 2B). When the ectodermal region was derived from a Cetn2 morphant, morphological extension was somewhat suppressed but notochordal tissue formed (Fig. 3C). Similar results were obtained using mesoderm only explants. Again, control explants displayed an extended morphology (Fig. 3D) and formed notochordal tissue (Fig. 3I). Cetn2 morphant explants failed to elongate (Fig. 3E) or form notochord (Fig. 3J). The morphological extension and notochord phenotypes displayed by Cetn2 morphant explants were rescued by Cetn2 (Fig. 3G,K) but not by Cetn3 (Fig. 3L) RNA injection. The Cetn2 morpholino elongation defect was similar to that seen in explants derived from embryos injected with RNA encoding a dominant negative form of FGF Receptor 1 (dnFGFR1)(Fig. 3F). That the Cetn2 morphant phenotype involves effects on FGF signaling was further suggested by the fact that the phenotype could be rescued by the injection of FGF8 RNA (Fig. 3M).

**Figure 4:**
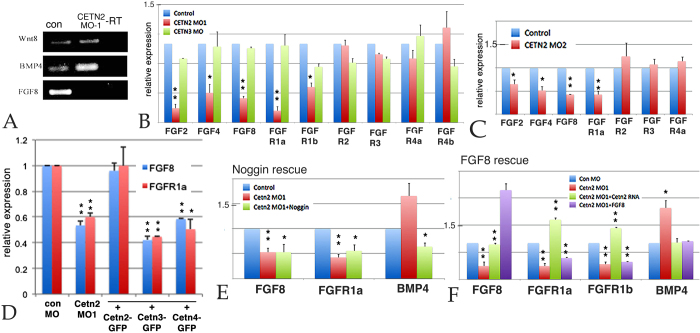
**A**: PCR analysis of control and Cetn2 MO embryos; there was a clear increase in BMP4 RNA, the disappearance of FGF8 RNA, and no apparent effect on Wnt8 RNA: B: qPCR analysis of Control, Cetn2MO1 and Cetn3MO embryos, injected in both cells of a two cell embryo and harvested at stage 11. **C:** A similar analysis carried out with the Cetn2MO2. **D**: qPCR analysis of embryos injected in both cells of a two cell embryo with Cetn2MO1 alone or together with Cetn2-GFP, Cetn3-GFP, or Cetn4-GFP RNAs (RNAs injected at 200 pg/side, total 400 pg/embryo; MOs injected at 10 ng/side, total 20 ng/embryo). While Cetn2-GFP RNA rescued the morphant phenotype Cetn3 or Cetn4 RNAs did not. **E**: qPCR analysis of embryos injected in both cells of two-cell embryos with Cetn2MO1 alone or together with Noggin RNA (200 pgs/embryo); Noggin reversed the morpholino effect on BMP4 RNA level but not the effect on FGF8 or FGFR1a RNA levels. **F**: qPCR analysis of embryos injected in both cells of a two cell embryo with Cetn2MO1 alone or together with either Cetn2-GFP or FGF8 RNAs (200 pgs/embryo); Cetn2 RNA rescued the Cetn2 morpholino’s effects on RNA levels, FGF8 reversed the effect on BMP4 RNA, but not the effects on FGFR1a RNAs. Levels of statistical significance indicated single * <0.05, double ** <0.001.

Together with retinoic acid, Wnt, FGF, and BMP are three of the most prominent signaling pathways involved in early embryonic patterning, including mesoderm/notochord formation^24^. A preliminary RT-PCR analysis of the levels of *Wnt8a*, *FGF8* and *BMP4a* RNAs in stage 11 control and *Cetn2* morphant embryos revealed a dramatic decrease in *FGF8* and an increase in *BMP4a* RNA levels, with no apparent change in *Wnt8a* RNA levels (Fig. 4A). This is a pattern of changing RNA levels quite distinct from that observed in ectodermal explants and whole embryos injected with morpholinos directed against either of two other cilia/basal body-associated proteins, Cby^22^ and EFHC1 (Zhao *et al*., in preparation). We confirmed this result using quantitative reverse transcription PCR (qPCR) of Cetn2 morphant embryos prepared by injecting either the MO1 or MO2 Cetn2 morpholinos. These morpholinos decreased the levels of *FGF2*, *FGF4*, *FGF8*, *FGFR1a*, and *FGFR1b* RNAs but produced no significant change in *FGFR2*, *FGFR3*, or FGFR4a RNAs. The *Cetn3* MO did not change the level of any *FGF* or *FGFR* RNA examined (Fig. 4B,C). In rescue studies, co-injection of Cetn2-GFP RNA could return the levels of FGF8 and FGFR1a to control levels, while Cetn3-GFP and Cetn4-GFP RNAs could not (Fig. 4D).

FGF and BMP have been found to regulate each other’s expression in the early embryo^25^. To determine whether the effects of Cetn2 morpholinos were direct or indirect, we injected RNA encoding the BMP antagonist Noggin^26^ into Cetn2 morphant embryos. Noggin RNA reduced the Cetn2 MO induced increase in the level of *BMP4a* RNA, but did not increase the level of *FGF8* RNA (Fig. 4E). In contrast, the injection of *FGF8* RNA reduced the Cetn2 MO induced increase in the level of *BMP4a* RNA, but failed to rescue the levels of *FGFR1a* RNA (Fig. 4F). This suggests that Cetn2 plays a direct role in the regulation of FGF8 and FGFR1a/b, while its effects on *BMP4a* RNA levels are indirect and the result of changes in FGF signaling.

**Figure 5:**
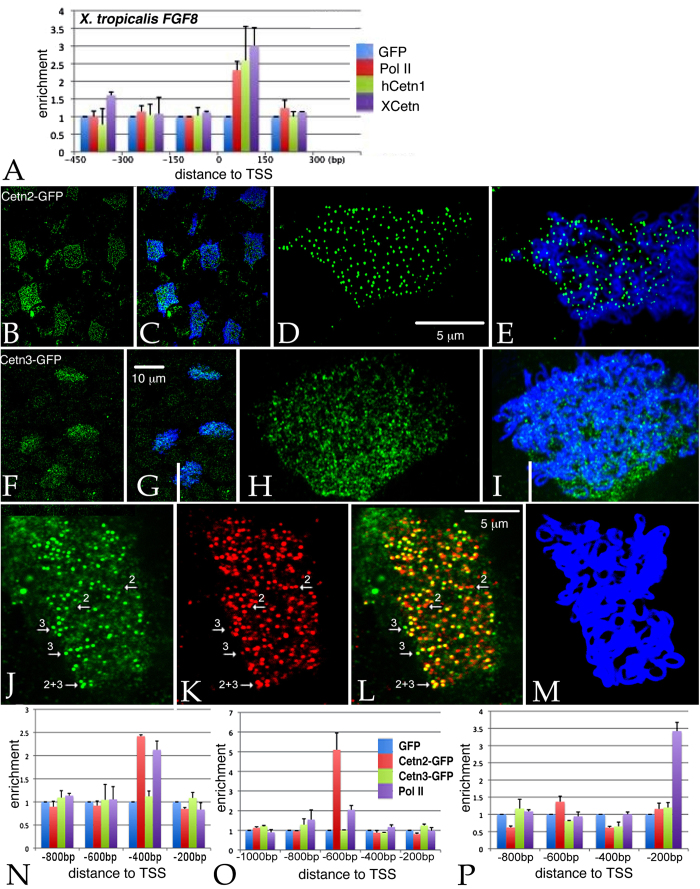
**A**: Unmaninpulated *X. tropicalis* embryos were isolated at stage 11, subjected to ChIP using 2 μg of either anti-GFP antibody (as control), anti-*Xenopus* Cetn antibody, anti-human Cetn1 antibody, or anti-Pol II antibodies. Isolated embryonic DNA was analyzed by qPCR using primers directed against the FGF8 promoter region. The distance from the transcription start site (TSS) is noted. For similar studies in *X. laevis*, we first characterized the behavior of the myc-Cetn2-GFP and myc-Cetn3-GFP polypeptides in ectodermal explants. Fertilized eggs were injected with encoding either myc-Cetn2-GFP RNA (**B-E**), myc-Cetn3-GFP RNA (**F-I**), or both Cetn2-RFP and myc-Cetn3-GFP RNAs (**K-M**)(each RNA injected at 200 pg/embryo). Ectodermal explants were isolated at stage 9 and fixed at stage 18. Immunofluorescence staining was performed using both anti-GFP and anti-AAT antibodies; scale bar in part **G** indicates 10 μm for parts **B**,**C**,**F** and **G**. Scale bar in parts **D & L** indicates 5 μm for parts **D**,**E**,**H**,**I** and **J-M.** Confocal images were taken at either 40X (**B,C,F,G**) or 100X (**D,E,H,I,J-M**) magnification. It is readily apparent that both myc-Cetn2-GFP and myc-Cetn3-GFP polypeptides accumulate in ciliated cells. In explants expressing both myc-Cetn3-GFP (**J**) and Cetn2-RFP (K; overlap in panel **L**, panel **M** is ATT staining), there was both extensive overlap in the localization of Cetn2 and Cetn3 polypeptides (arrow marked “2 + 3”), as well as sites where one or the other predominates (arrows marked either “2” or “3”). For ChIP studies in *X. laevis*, both blastomeres of 2-cell stage embryos were injected with RNAs encoding either GFP, myc-Cetn2-GFP, or myc-Cetn3-GFP; uninjected embryos were used as a control. Embryos were harvested at stage 11. GFP antibody was used to immunoprecipitate the injected embryos and Pol II antibody was used to immunoprecipitate the uninjected embryos. qPCR analysis was performed to check protein binding to the FGFR1a (**N**), FGF8 (**O**) and BMP4a (**P**) promoter regions.

Given Cetn2’s established presence in the nucleus (see above) and the effects of the Cetn2 morpholinos on *FGF8* and *FGFR1a* RNA levels, it seemed plausible that Cetn2 might directly influence the expression of these genes through interactions with chromatin. We initially examined the *FGF8* gene in *X. tropicalis* using anti-XlCetn, anti-HsCetn1 (which preferentially reacts with Cetn2 over Cetn3 - see above), and anti-RNA polymerase II antibodies. We found that Cetn co-localized with polymerase at these loci (Fig. 5A). Further experiments in *X. laevis* used RNAs encoding GFP-tagged forms of *X. laevis* Cetn2a, Cetn3l, and Cetn4 (see Methods). In ectodermal explants derived from RNA injected fertilized eggs, myc-Cetn2-GFP (Fig. 5B,C) and Cetn4-GFP (**SupFig. 4**) polypeptides preferentially accumulated in ciliated cells and localized to the basal body region of cilia (Fig. 5D,E). myc-Cetn3-GFP was also found to accumulate preferentially in ciliated cells (Fig. 5F,G) and was localized to basal bodies (Fig. 5H,I), but its localization was not quite as cilia-specific as that observed for Cetn2. That said, in ectodermal explants expressing (from injected RNA) both a C-terminally RFP-tagged Cetn2 (Cetn2-RFP) and myc-Cetn3-GFP, we saw regions of high overlap, as well as regions of distinct Cetn2 and Cetn3 accumulation (Fig. 5J–M). In chromatin immunoprecipitation (ChIP) experiments, myc-Cetn2-GFP but not myc-Cetn3-GFP or GFP alone, co-localized with Pol II on the *FGF8* and *FGFR1a* genes (Fig. 5N,O); neither Cetn localized with Pol II on the *BMP4a* gene (Fig. 5P), as expected for an indirect target of Cetn2-mediated regulation.

Our work reveals a new and unexpected role for Cetn2 as a transcriptional regulator of a subset of FGF and FGFR genes. The fact that Cetn2 was found to co-localize with Pol II at some, but not all genes suggests that its promoter association is dependent upon the presence of other proteins. In addition to its nuclear role as part of the XPC DNA repair complex, Cetn2 has been reported to be an integral component of the Trex2 complex, which appears to interact with both nuclear pores and RNA polymerase and has been implicated in the nuclear export of mRNAs^27^,^28^. This suggests that the Cetn2 morphant phenotype could, in part, involve changes in Trex2 function or other, as yet unidentified interactions. To resolve this issue, we are currently in the process of identifying Cetn2-associated proteins in *Xenopus* and other systems.

The role of Cetn2 as a regulator of mesodermal differentiation, as revealed by notochord formation, in *Xenopus* early embryonic development may seem at odds with the reported phenotypes of morpholino treated Zebrafish embryos and Cetn2 null mice. In the Zebrafish *Danio rerio*, depletion of Cetn2 led to a ciliopathy-related cyst formation phenotype^11^. A syndromic ciliopathy, including dysosmia and hydrocephalus was reported in Cetn2 null mice^12^. We anticipate that the phenotypic differences between these three vertebrates can be attributed to differences in developmental mechanisms and Cetn2-containing complexes, the patterns of centrin gene expression during embryonic development, and perhaps some level of partial redundancy between the centrin genes, although neither Cetn3 or Cetn4 RNAs rescued the Cetn2 morphant gene expression phenotype. That said, a role of Cetn2 in the regulation of gene expression, and its integral role in the functions of XPC, Trex2, and perhaps other complexes, indicates the need for more subtle analyzes of the physiological roles of Cetn2 in particular and Cetns as a class of proteins.

## Materials and Methods

### Embryos, their manipulation and analysis

*X. laevi*s embryos were staged, and explants were generated, following standard procedures^29^. Capped mRNAs were transcribed from linearized plasmid templates using mMessage mMachine kits (Ambion) following manufacturer’s instructions. At the two-cell stage embryo injections were directed equatorially. As an injection tracer, we routinely included RNAs (150 pgs/embryo) encoding either β-galactosidase, green fluorescent protein (GFP) or GFP-CAAX, which is membrane-associated. In the case of GFP/GFP-CAAX RNA injection, embryos were examined at stage 10-11 by fluorescent microscopy to confirm the accuracy of injection. RNA isolation, cDNA synthesis, RT-PCR and qPCR analyses were carried out as described previously^22^,^23^. Real-time (quantitative) PCR was carried out using a Mastercycler Epgradient Realplex device (Eppendorf). PCR reactions were set up using DyNAmo SYBR Green qPCR kits (Finnzymes). Each sample was normalized to the expression level of ornithine decarboxylase (ODC). The cycling conditions used were: 95°C for 5 minutes; then 40 cycles of 95°C for 15 seconds, 56°C for 15 seconds, 60°C for 30 seconds. The ΔΔCT method was used to calculate real-time PCR results. The primers used for RT-PCR analysis were Ornithine decarboxylase (ODC) [U 5′-CAG CTA GCT GTG GTG TGG-3′ D 5′-CAA CAT GGA AAC TCA CAC-3′]; Wnt8a [U 5′-TGA TGC CTT CAC TTC TGT GG-3′ D 5′-TCC TGC AGC TTC TTC TCT CC -3′]; BMP4 [U 5′-TGG TGG ATT AGT CTC GTG TCC -3′ D 5′-TCA ACC TCA GCA GCA TTC C -3′]; Noggin [U 5′-AGT TGC AGA TGT GGC TCT-3′ D 5′-AGT CCA AGA GTC TCA GCA -3′]; FGF8 [U 5′-TGG TGA CCG ACC AAC TAA GC D 5′-CGA TTA ACT TGG CGT GTG G ]; FGF2 [U 5′- AGA GGC TCT ACT GCA AGA ACG-3′ D 5′- TTC CTT CAT GGC AAG GTA GC-3′]; FGF4 [U 5′-GCA TGC CGT TCT CTT CTT CC -3′ D 5′-ACG TCG CAG TCT CTT GAT GC -3′]; FGFR1a [U 5′-GCG CAT TGG TGG ATA TAA GG -3′ D 5′- AGA CCG GCT TGT AGG ATT GG-3′]; FGFR1b [U 5′-GCG CAT TGG TGG ATA TAA GG -3′ D 5′-GAG ACC GGC TTG TAG GAT AGG -3′]; FGFR2 [U 5′- TCT GCA TGG TAG TGG TCT GC-3′ D 5′- CAG GAG TCG TGT TGT GAT CC-3′]; FGFR3 [U 5′- ACC AAG TGG TTC AAG GAT GG D 5′- TCG TCA TCC TCA TCA TCA CC]; FGFR4a [U 5′-ACA GTC AAG TTC CGC TGT CC -3′ D 5′-GCT GCC AAC TCT GTT CTC TAC C -3′]; and FGFR4b [U 5′-ACA CTG GAG CCT GGT AAT GG -3′ D 5′-GCT ACC TAC ACG TGC TGT GG -3′].

### Morpholinos and plasmids

Cetn coding sequences were isolated from maternal RNA by RT-PCR and subcloned into pCS2 plasmids, following protocols used previously to isolate Cby coding sequences^22^; where indicated such constructs had N-terminal myc and C-terminal GFP sequences^30^. We also generated plasmids that encode Cetn-GFP chimeras that either perfectly matched or were maximally mismatched to their respective morpholinos. Plasmids encoding either N- or C-terminally tagged Cetn2-RFP were obtained from Sergie Sokol (Mount Sinai School of Medicine) and John Wallingford (U. Texas, Austin). Plasmids encoding FGF8 and a dominant-negative form of FGFR1 were supplied by Enrique Amaya (U. Manchester). Morpholinos against *X. laevis* centrins were designed and synthesized by Gene Tools. These included two non-overlapping morpholinos against Cetn-2a [MO1 5′CTTGTAGTTAGAAGCCATATCACAC 3′ and MO2 5′TGCACACACCAACCTTCGACCTCGC 3′], a Cetn-3l morpholino [5′CATCAGTCCTCACAGCCAGGCTCAT 3′], and a Cetn-4 morpholino [5′CTGGTTTACGCAGAACAGAGGCCAT 3′](supplemental figure. 2). In each case, a NIH-Blast search of the morpholino sequence revealed only a single hit in *X. laevis*, namely the targeted Cetn RNA. A plasmid encoding membrane-bound GFP, GFP-CAAX, was supplied by Kristen Kwan (U. Utah). Statistical analyses were based on values expressed as mean ± standard deviation and analyzed using the unpaired student’s t-test. p < 0.05 was considered as significant in all analyses.

### Immunocytochemistry and imaging

Embryos were fixed and stained as described by Dent *et al.*^31^. The mouse monoclonal anti-acetylated α-tubulin antibody was used to visualized ciliated cells^32^. The rabbit anti-Xenopus Cetn antibody (anti-XlCetn) was supplied by Sergie Sokol^20^. The rabbit anti-human Cetn1 antibody (anti-HsCetn1) was purchased from Sigma. The anti-HsCetn1 antibody is directed against the C-terminal 15 amino acids of Cetn1; a similar sequence exists at the C-termini of *X. laevis* Cetn2 (13/15 identical) and Cetn4 proteins (12/15 identical), but is absent from the Cetn3 (5/15 identical)(data not shown). Notochord was visualized using the mouse monoclonal anti-keratin sulfate antibody MZ15 obtained from the Developmental Studies Hybridoma Bank^33^. Immunoperoxidase-stained explants were bleached before staining while immunofluorescently-labeled embryos were not. Fluorescent images were collected using a Zeiss 510 Confocal Laser Scanning Microscope.

### ChIP studies

Chromatin immunoprecipitation (ChIP) was performed following the protocol described in Blythe *et al.*^34^. Briefly, at stage 11 uninjected embryos, or embryos injected with GFP, CETN2-GFP, or CETN3-GFP RNAs were cross-linked with 1% formaldehyde for 30 minutes; cross-linking was stopped with a 10 min wash in 0.125M Glycine/PBS, followed by three washes in PBS. Samples were resuspended in RIPA buffer (50 mM Tris-HCl, pH 7.4, 1% NP-40 (Sigma I3021), 0.25% Na-Deoxycholate,150 mM NaCl, 1 mM EDTA, 0.1% SDS, 0.5 mM DTT, 5mM Na-Butyrate, Protease Inhibitor Cocktail (Sigma P8340), Phosphatase Inhibitor Cocktail I (SigmaP2850), Phosphatase Inhibitor Cocktail II (Sigma P5726). Chromatin was sonicated on ice to an average size of 200-500 base pairs using a Bioruptor (Diagenode) . Immunoprecipitation was performed using Dynabeads Protein G (Life Technologies) coupled to 2 μg GFP antibody (ICL, Inc) or Pol II antibody (Diagenode) at 4°C overnight. The bound chromatin was eluted and cross-links were reversed at 65°C overnight. ChIP DNA was extracted with phenol-chloroform and qPCR was performed. Below is a list of the primers used in ChIP studies. *X. tropicalis* CHIP primers: Fgf8 [primer 1 U 5′- TGA CTT TGC GCT CTG ACT TT-3′ D 5′- AAA AGA AAC AGC CGA GAT GC-3′]; [primer 2 U 5′- GCA TCT CGG CTG TTT CTT TT -3′ D 5′- GAT AGT GAT GGG GAG AGC CT -3′]; [primer 3 U 5′- CAGGCTCTCCCCATCACTAT -3′ D 5′- GAG TAG AAA CAC GCT GCA CT-3′]; [primer 4 U 5′- CCT CTC TTC CAG ACT CGG CT -3′ D 5′- GGA GTC GGA TTG CAG TGG AG-3′]; [primer 5 U 5′-TGA GCT ACA TCA CCT CCA TC-3′ D 5′-GAA GAG AAG GTC CAG TTA GCA -3′]. X. laevis CHIP primers: FGFR1a [primer 1 U 5′-ACA GAG TGG CAA TTA TAC AGA GG -3′ D 5′-CAC CTC ACA GCC ACA ATA CC -3′]; [primer 2 U 5′-GAG AGG CTG TGA GCA TAA TGG-3′ D 5′-TGC TAC TAT AGG CAC CAT CAC C-3′]; [primer 3 U 5′-GGA GAG AGA TGG TGC CTA TGG -3′ D 5′-TCT TCC TAC TAC ACT TGC TGT CC-3′]; [primer 4 U 5′-TGG TGC CTA TAG TAG CAG TGG -3′ D 5′-CTC CAG AGC ACA AGC ATG G-3′]; FGF8 [primer 1 U 5′-CAC TCA CAC TGT GTC TCT CAG G -3′ D 5′-CCG GCC AAT AAC ACT AGA AGC-3′]; [primer 2 U 5′-TCA GCT CAG ACA CAC CAA GC -3′ D 5′-TTC TCT CTC TCT CTC GCT TCC -3′]; [primer 3 U 5′- TCA GCT CAG ACA CAC CAA GC -3′ D 5′- CAA GGA GGC GAG TTA CTT CC -3′]; [primer 4 U 5′- AGT AAC TCG CCT CCT TGT CG -3′ D 5′- GCC TCT CAA GAG CAA GAT GC -3′]; [primer 5 U 5′- GAC AGT AGC GCA ACA CTC G -3′ D 5′- GCC TCT CAA GAG CAA GAT GC -3′]; BMP4a [primer 1 U 5′- TTG GCT GTC AAG AAT CAT GG -3′ D 5′- CAG CAG GAA GTA GCC AGA GC -3′]; [primer 2 U 5′- ACA CGG CTC TGG CTA CTT CC -3′ D 5′- AGC CTG GCC AAT GAA TGC -3′]; [primer 3 U 5′- GGA CAT ATC GCA GGC TAT CG -3′ D 5′- TCA GAT AGT CAC CGC CAT CC -3′]; [primer 4 U 5′- GAG ACG CTC TCA GTC AGA TTA GC -3′ D 5′- CAA GGA CAG TTC CAC AGA GG -3′].

### Note added in proof

Recently, human centrin 2 has been shown to regulate primary cilia formation through controlling CP110^35^.

## Additional Information

**How to cite this article**: Shi, J. *et al*. Centrin-2 (Cetn2) mediated regulation of FGF/FGFR gene expression in *Xenopus.* *Sci. Rep.* **5**, 10283; doi: 10.1038/srep10283 (2015).

## Supplementary Material

Supplementary Information
