# Supplementary material for: Centrin-2 (Cetn2) mediated regulation of FGF/FGFR gene expression in Xenopus
Source: Sci Rep. 2015 May 27;5:10283. doi: 10.1038/srep10283 (PMC4650658; doi:10.1038/srep10283)

Supplemental Figures for:

Centrin-2 (Cetn2) mediated regulation of FGF/FGFR gene expression in *Xenopus*

Jianli Shi, Ying Zhao, Tyson Vonderfecht, Mark Winey & Michael W. Klymkowsky*

Supplemental figure 1 A: In both *X. laevis* and *X. tropicalis*, the structure of the gene originally called *Centrin*/*Centrin-1* is more like that of mouse and human *Cetn2s;* we refer to these *Xenopus* genes as *Cetn2.* There does not appear to be a Cetn1-like gene in the Xenopus genome. **B**. A comparison of the X. laevis Cetn2 protein to human Cetn1 and Cetn2 proteins.

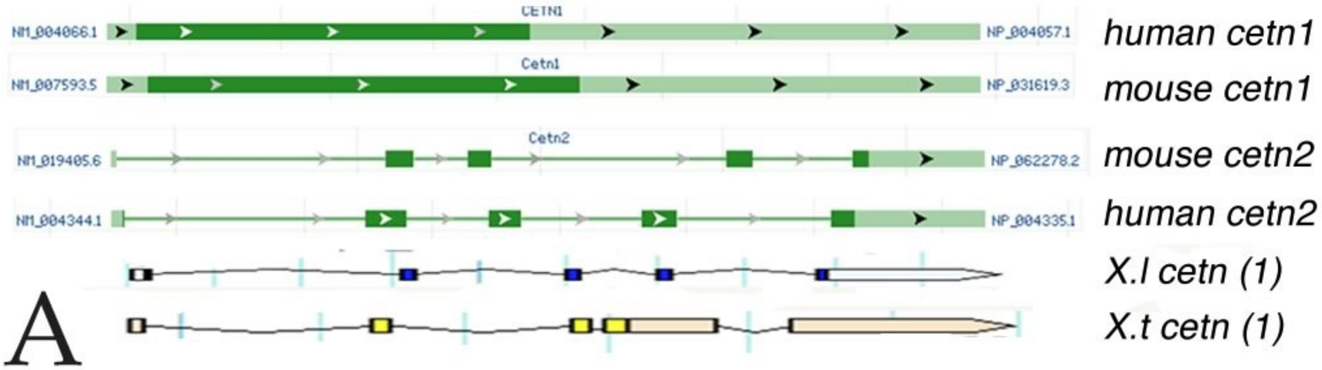

B

```
hsCETN1   1    GF     AS G KR VA  P    D    V        V  S
XlCETN    1  MASNYKKPSLGVTTQRKKPVPKTELTEEQKQEIREAFDLFDTDGTGTIDVKELKVAMRAL
hsCETN2   1      F  ANMASSS   RMS  P                  A

hsCETN1  61      R   M    SEV R      S N  LAV     S   T    L
XlCETN   61  GFEPKKEEIKKMIADIDKEGTGKIAFSDFMCAMTQKMAEKDSKEEIMKAFRLFDDDETGK
hsCETN2  61               SE        MN G  LTV     S   T    L   K

hsCETN1 121            -                            E           Y
XlCETN  121  ISFKNLKRVAKELGENLTDEELQEMIDEADRDGDGEVNEQEFLRIMKKTSLF
hsCETN2 121                                       S             Y
```

Supplemental Figures for:

Centrin-2 (Cetn2) mediated regulation of FGF/FGFR gene expression in *Xenopus*

Jianli Shi, Ying Zhao, Tyson Vonderfecht, Mark Winey & Michael W. Klymkowsky*

Supplemental figure 2: A. The morpholinos directed against the three *X. laevis* Cetns that are the focus of our studies are shown together with their target sequences. Cetn2 corresponds to the *Cetn2a* RNA; Cetn3 corresponds to the *Cetn3l* RNA. Not shown are the mismatches between the Cetn3 morpholino and the *Cetn3s* RNA (15 mismatched out of 25) and the mismatches between the Cetn2 MO2 morpholino and the *Cetn3s* RNA (the terminal 7 bases mismatched out of 25).

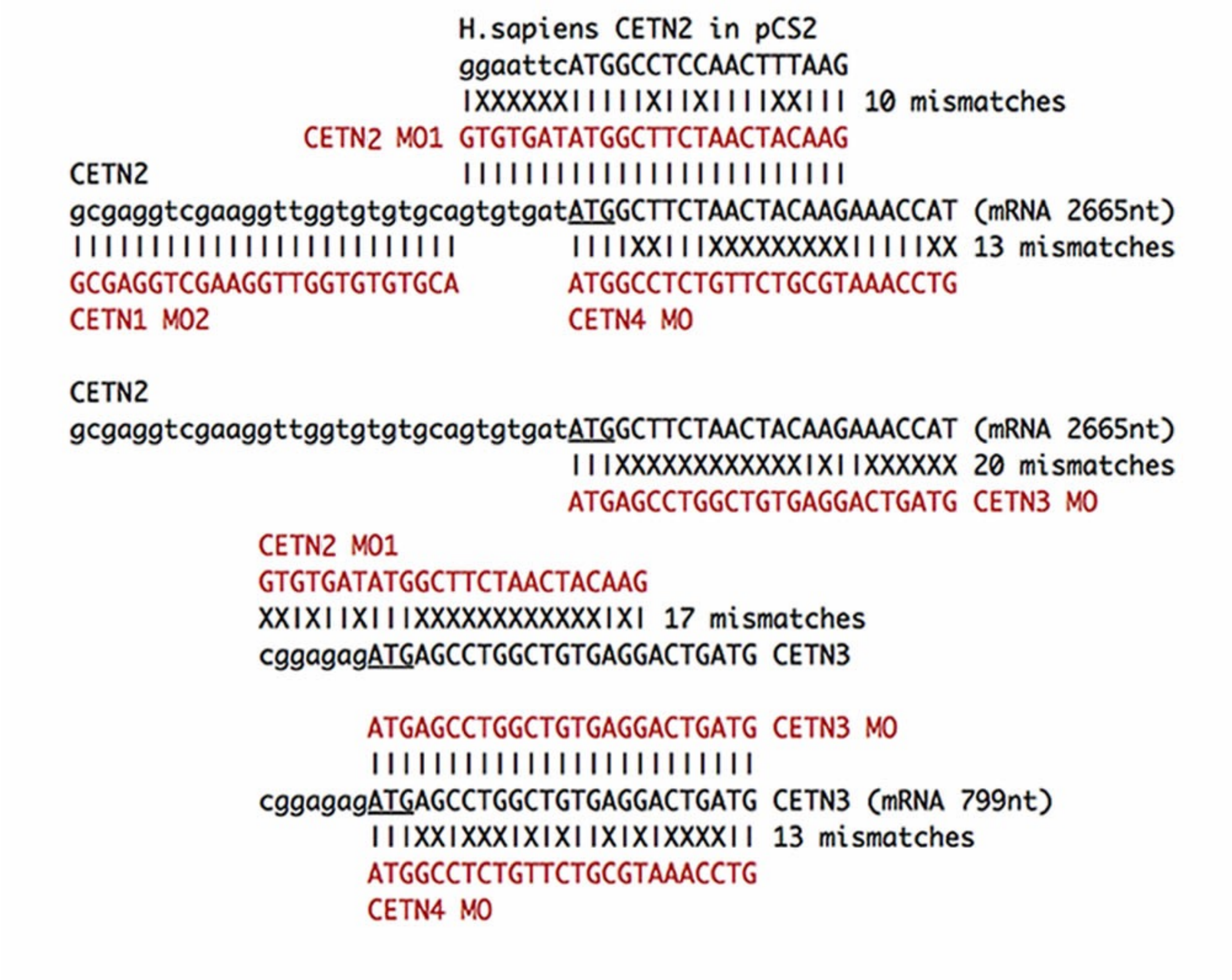

Supplemental Figures for:

Centrin-2 (Cetn2) mediated regulation of FGF/FGFR gene expression in *Xenopus*

Jianli Shi, Ying Zhao, Tyson Vonderfecht, Mark Winey & Michael W. Klymkowsky*

Supplemental Figure 3: Both blastomeres of two-cell embryos were injected with either control morpholino (not shown), Cetn2 MO1 (**B**), Cetn2 MO2 **(C**), Cetn3 MO (**D**), or Cetn4 MO (**E**)(20 ngs/embryo total injected), and compared with uninjected embryos (**A**)**.** The embryos were fixed at stage 39-40. To examine the roles of Cetns in ciliogenesis, both blastomeres of two cell stage embryos were injected with either control morpholino (**F**), Cetn2 MO1 (**G**), Cetn3 MO (**H**), Cetn2MO1 and Cetn4 MO (**I**), or Cetn2, 3, 4 MOs (**J**)(20ng/embryo). Ectodermal explants were isolated at stage 9 and fixed at stage 18. Immunofluorescence staining was performed with anti-XlCetn and anti-AAT antibodies to visualize ciliated cells. Confocal images were taken at 40X magnification. The scale bar in part J marks 20 µm for parts F-J. The results indicate the general trends, but a more quantitative analysis using semi-automated confocal microscopy based quantitation (as described in Shi et al., 2014) is planned.

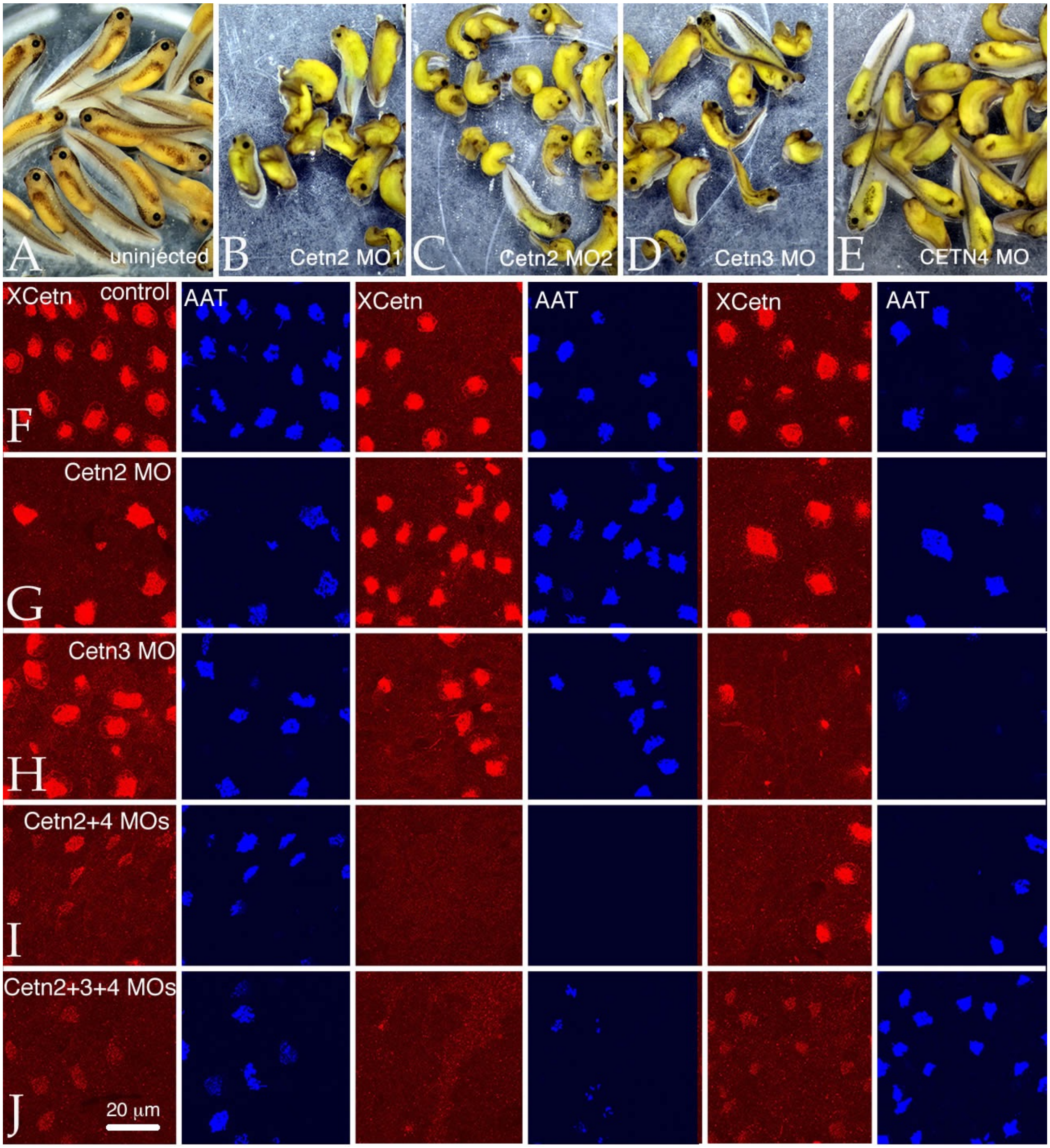

supplemental figure 3

Supplemental Figures for:

Centrin-2 (Cetn2) mediated regulation of FGF/FGFR gene expression in *Xenopus*

Jianli Shi, Ying Zhao, Tyson Vonderfecht, Mark Winey & Michael W. Klymkowsky*

Supplemental figure 4: To characterize the intracellular localization of Cetn4-GFP, both blastomeres of two cell embryos were injected with Cetn4-GFP RNA (200 pg/embryo) Ectodermal explants were isolated at stage 9 and fixed at stage 18. Immunofluorescence staining was performed with anti-GFP (**A,D**) and anti-AAT (**B,E**) antibodies to visualize ciliated cells (parts **C** and **F** are overlap images). Confocal images were taken at 40X magnification for parts **A-C** and at 100X for parts **D-I**. Scale bar in part **A** marks 10 µm for parts **A-C**, while scale bar in part **D** marks 5 µm for parts **D-F**.

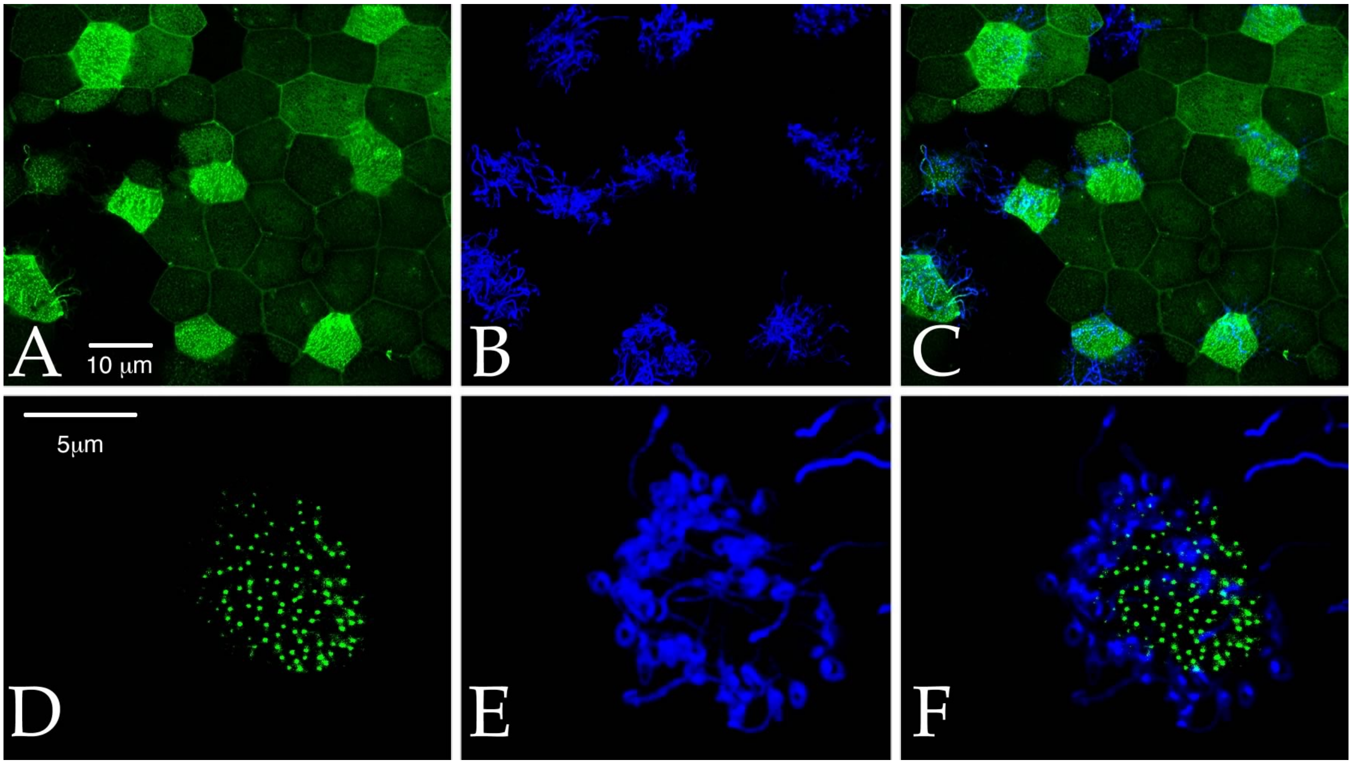

Supplement: Supplementary Information [file srep10283-s1.pdf]
